# A Further Case Supporting *PDCD6IP* as the Gene Responsible for a Neurodevelopmental Disorder With Microcephaly

**DOI:** 10.1111/cge.70025

**Published:** 2025-07-16

**Authors:** Alfonso Manuel D'Alessio, Annalaura Torella, Manuela Morleo, Vincenzo Nigro, TUDP Study Group, Nicola Brunetti‐Pierri

**Affiliations:** ^1^ Telethon Institute of Genetics and Medicine (TIGEM) Pozzuoli Italy; ^2^ Department of Translational Medicine University of Naples “Federico II” Naples Italy; ^3^ Scuola Superiore Meridionale, Genomics and Experimental Medicine Program University “Federico II” Naples Italy; ^4^ Department of Precision Medicine University “Luigi Vanvitelli” Naples Italy

**Keywords:** genetic disease, intellectual disability, microcephaly, multiple fractures, neurodevelopmental disorder, PDCD6IP, rare disease

## Abstract

Summary of clinical and molecular findings in patients with biallelic variants in *PDCD6IP*.

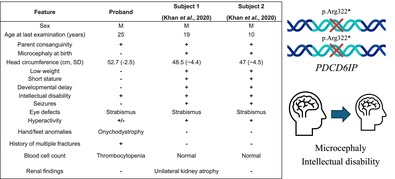

A homozygous truncating variant in *PDCD6IP* was recently reported in two siblings with primary microcephaly, intellectual disability, and short stature from a consanguineous Saudi family [1]. Although additional unrelated cases are lacking, microcephaly observed in *Pdcd6ip* knockout mice [2, 3] and *pdcd6ip* knockdown zebrafish [4] supports the pathogenicity of PDCD6IP loss‐of‐function variants. *PDCD6IP* encodes a multifunctional cytosolic scaffold protein that regulates Endosomal Sorting Complexes Required for Transport (ESCRT), which are involved in intracellular trafficking, membrane remodeling, exosome biogenesis, and cytokinesis [5].

Here, we report an unrelated individual with mild intellectual disability and microcephaly carrying a homozygous nonsense variant in *PDCD6IP*.

The patient, first of four children born to first‐cousin parents, was delivered at term following an uneventful pregnancy. His 21‐ and 4‐year‐old sisters were healthy; his 15‐year‐old sister had mild learning difficulties. His mother interrupted one pregnancy due to multiple fetal malformations and had a spontaneous miscarriage.

At birth, his weight was 3450 g (−0.23 SD), his length 50 cm (−0.44 SD) and his OFC 34 cm (−0.78 SD). Perinatal events were normal except for a clavicle fracture. He said his first words at 8 months of age and walked independently at 18 months of age. When he was a child, he had six fractures involving ankles, knees, and arms following low‐intensity impacts. He underwent surgery for bilateral *pes planus*, *hallux valgus*, and hammer toes. Since he attended infancy school, he showed hyperactivity and learning difficulties, and he completed high school with the assistance of a dedicated teacher. At the age of 21, he was able to read and to perform simple calculations, but he had trouble performing complex tasks. He worked under his father's supervision in logistics, handling, and unloading goods.

At the age of 25 years, he showed a sloping forehead and a prominent columella. His parameters were: weight 96.9 kg (+2.3 SD), height 173 cm (−0.6 SD) OFC 51.6 cm (−3.1 SD). Bilateral onychodystrophy of the halluces and right hallux valgus were observed.

He was found to have persistent isolated thrombocytopenia (lowest value: 50 × 10^9^/L) with no history of bleedings or petechiae. The mean platelet volume was slightly increased (13.9 fL, ULN: 10 fL). A bone marrow biopsy ruled out malignancies and showed hyperplastic megakaryocytes, and no abnormalities in erythroid, lymphoid, and myeloid lineages. Moreover, he had a persistent mild increase in serum CPK (highest value: 460 IU/L) without muscle fatigue, weakness, or cramps. One of his sisters was also found to have thrombocytopenia and onychodystrophy, while no members of the family showed alterations in serum CPK. Brain MRI only showed a thin corpus callosum. Auditory evoked potentials, echocardiogram, abdomen, and thyroid ultrasonographies showed no abnormalities. Standard karyotype performed on peripheral blood and bone marrow aspirate was normal. Moreover, chromosome breakage test, Fragile‐X testing, array‐CGH, and clinical exome sequencing showed no alterations. Exome sequencing (ES) showed a homozygous nonsense variant c.964C>T p.(Arg322*) in the *PDCD6IP* gene (NM_013374.6). Both parents, his 21‐year‐old and 15‐year‐old sisters, were heterozygous carriers of the variant. The variant has low frequency in gnomAD (allele frequency: 4.34 × 10^−6^), has never been observed in homozygous state, and is predicted as pathogenic according to ACMG criteria (PVS1, PM2, and PM3). No other variants detected in the ES were consistent with the phenotype.

Unlike previously reported familial cases with *PDCD6IP* biallelic variants, the individual described herein had acquired microcephaly, mild CPK elevation, and no epilepsy (Table 1). The proband also exhibited chronic thrombocytopenia, shared with an unaffected carrier sister, suggesting it is unrelated to the PDCD6IP defect and a history of multiple fractures. No variants in other genes explaining these clinical features were detected.

**TABLE 1:** Clinical features of the three patients known so far with *PDCD6IP*‐related disorder.

Feature	Proband	Subject 1 (Khan et al. [1])	Subject 2 (Khan et al. [1])
Sex	M	M	M
Age at last examination (years)	25	19	10
Parent consanguinity	+	+	+
Microcephaly at birth	−	+	+
Head circumference (cm, SD)	52.7 (−2.5)	48.5 (−4.4)	47 (−4.5)
Low weight	−	+	+
Short stature	−	+	+
Developmental delay	−	+	+
Intellectual disability	+	+	+
Seizures	−	+	+
Eye defects	Strabismus	Strabismus	Strabismus
Hyperactivity	±	+	+
Hand/foot anomalies	Onychodystrophy	−	−
History of multiple fractures	+	−	−
Blood cell count	Thrombocytopenia	Normal	Normal
Renal findings	−	Unilateral kidney atrophy	−

This additional case of biallelic *PDCD6IP* variants further supports its role in a novel neurodevelopmental disorder with microcephaly. All three known cases involve consanguineous families, indicating these variants are likely extremely rare in the general population. As *PDCD6IP* is not yet classified as a disease‐causing gene, its pathogenic variants may be underrecognized. Our case highlights the importance of including *PDCD6IP* as a disease‐causing gene in patients with intellectual disability and microcephaly.

## Conflicts of Interest

The authors declare no conflicts of interest.

## Peer Review

The peer review history for this article is available at https://www.webofscience.com/api/gateway/wos/peer‐review/10.1111/cge.70025.

## Data Availability

The data that support the findings of this study are available on request from the corresponding author. The data are not publicly available due to privacy or ethical restrictions.
